# Persistence of HPV infection and risk of high-grade cervical intraepithelial neoplasia in a cohort of Colombian women

**DOI:** 10.1038/sj.bjc.6604972

**Published:** 2009-03-17

**Authors:** N Muñoz, G Hernandez-Suarez, F Méndez, M Molano, H Posso, V Moreno, R Murillo, M Ronderos, C Meijer, Á Muñoz

**Affiliations:** 1 Subdirección de Investigaciones y Salud Publica, Instituto Nacional de Cancerología, Bogotá, Colombia; 2 Escuela de Salud Pública, Universidad del Valle, Cali, Colombia; 3 Cancer Epidemiology Service, Instituto Catalán de Oncología, Barcelona, Spain; 4 Department of Pathology, Vrije Universiteit Medical Center, Amsterdam, The Netherlands; 5 Department of Epidemiology, Johns Hopkins Bloomberg School of Public Health, Baltimore, MD, USA

**Keywords:** human papillomavirus, persistence, risk, cervical intraepithelial neoplasia

## Abstract

Little is known about the dynamics of human papillomavirus (HPV) infection and subsequent development of high-grade cervical intraepithelial neoplasia (CIN2/3), particularly in women >30 years of age. This information is needed to assess the impact of HPV vaccines and consider new screening strategies. A cohort of 1728 women 15–85 years old with normal cytology at baseline was followed every 6 months for an average of 9 years. Women with squamous intraepithelial lesions were referred for biopsy and treatment. The Kaplan–Meier method was used to estimate the median duration of infection and Cox regression analysis was undertaken to assess determinants of clearance and risk of CIN2/3 associated with HPV persistence. No difference in the likelihood of clearance was observed by HPV type or woman's age, with the exception of lower clearance for HPV16 infection in women under 30 years of age. Viral load was inversely associated with clearance. In conclusion, viral load is the main determinant of persistence, and persistence of HPV16 infections carry a higher risk of CIN2/3.

Cervical human papillomavirus (HPV) infection is the necessary, but not a sufficient, cause of cervical cancer (Walboomers *et al*, 1999; Muñoz *et al*, 2003). Only a small fraction of those with persistent HPV infections develop cervical cancer and its immediate precursors (Schiffman and Kjaer, 2003). Although persistent HPV infection has been considered a prerequisite for cervical cancer (Bosch *et al*, 2008), there is no consensus on its definition. Most investigators define it as detection of the same HPV type or group of types on two consecutive visits, but these could be from 4 months up to 5–7 years apart (Schiffman and Kjaer, 2003; Castle *et al*, 2005). A recent review of problems with this approach suggests restricting analyses to incident infections, and considering the duration of the infection rather than the number of positive tests (Woodman *et al*, 2007). Agreement on definition will facilitate comparisons of results from studies, on end points in vaccine trials and screening policy recommendations. Some recent reports cast doubts on the need for persistence for progression to high-grade squamous intraepithelial lesions (HSIL) or intraepithelial neoplasia (CIN2/3) (Schiffman *et al*, 2005; Winer *et al*, 2005). Moreover, most cohort studies of cervical HPV infections have been in young women (Ho *et al*, 1998; Moscicki *et al*, 2001; Woodman *et al*, 2001; Brown *et al*, 2005; Winer *et al*, 2005), one suggesting that HSIL is often an early manifestation of HPV infection in young women (Winer *et al*, 2005). We present results on clearance of HPV incident infection and its determinants and estimate the risk of progression to CIN2/3 in a cohort of young and older Colombian women followed at regular scheduled visits. We propose a new definition of persistence based on duration of infection, and compare it with the traditional definition based on detection of HPV DNA in two consecutive visits.

## Materials and methods

Between November 1993 and November 1995, the National Cancer Institute of Colombia (INC) invited 2200 women aged 15–85 years to participate in a prospective study (Molano *et al*, 2002a), previously approved by the INC ethics committee. All study participants signed an informed consent form in compliance with the clinical research guidelines. The 2200 women were randomly selected from four low-income health districts of Bogotá who had consulted cervical cancer screening centres or family planning clinics. Methods of recruitment and data collection have been described elsewhere (Molano *et al*, 2002a, 2002b). Briefly, eligible women were those residing in Bogotá, without history of cervical neoplasia, conisation or hysterectomy, willing to participate and who signed an informed consent form. At study entry, participants responded to a questionnaire on risk factors for cervical cancer and underwent a pelvic examination with collection of cervical cells for cytology and HPV detection. Follow-up visits were scheduled every 6 months until March 2004. At each visit, a questionnaire on lifestyle and sexual behaviour was used, a pelvic examination was performed and cervical specimens were collected using Ayre spatulas and endocervical brushes for cytology and HPV detection. The specimens were eluted in phosphate-buffered saline +0.05% thiomersal. Colposcopic examination was performed in all women with repeated diagnosis of low-grade squamous intraepithelial lesions (LSIL) or with cytological evidence of HSIL. Colposcopically guided cervical biopsies were performed in women with cytological or colposcopic evidence of HSIL.

From the 2200 women invited to participate, 53 (2.4%) refused and 8 (0.4%) were ineligible (due to mental illness, hysterectomy or history of cervical cancer) leaving 2139 women. For this analysis, we only included women with normal Pap smear results and a valid HPV DNA sample at baseline with at least two visits during follow-up. If DNA results were not available for the first visit (because no scrape was available or due to failure to amplify the *β*-globin gene), the second visit was used as baseline.

Human papillomavirus presence and type was assessed by Southern blot hybridisation of GP5+/bioGP6+ PCR products using a general probe of specific DNA fragments from cloned DNA of six HPV types, and by the GP5+/GP6+ PCR enzyme immunoassay (PCR-EIA) as previously described (Jacobs *et al*, 1997; Molano *et al*, 2002a). For the EIA, HPV-positive samples were first subjected to a group-specific analysis using cocktail probes for high-risk (HR) and low-risk (LR) HPVs before individual typing was performed. The HR cocktail probe consisted of oligoprobes for HPV16, 18, 31, 33, 35, 39, 45, 51, 52, 56, 58, 59, 66 and 68; the LR HPV probe consisted of oligoprobes for HPV6, 11, 26, 34, 40, 42, 43, 44, 53, 54, 55, 57, 61, 70, 71, 72, 73, 81, 82 (MM4 and IS39 subtype), 83, 84 and CP6108. Types 73, 82, 26 and 53 were classified as HR (Muñoz *et al*, 2003). During follow-up, GP5+/GP6+ PCR reverse line blot (PCR-RLB) analysis was validated and used to type the 37 different HPV types detected by PCR-EIA (van den Brule *et al*, 2002). In this validation process, some samples from our cohort were tested using both techniques and 96% agreement was observed between the PCR-RLB analysis and the PCR-EIA assay. Specimens from the first four visits were typed with PCR-EIA and those from subsequent visits were typed with PCR-RLB. PCR-EIA was used to assess viral load in all visits. This is a semiquantitative method based on the linear relationship between the amount of DNA and the optical density (OD) in the range of 10–10^6^ genome equivalents (Jacobs *et al*, 1997).

Pap smears were read by a cytotechnologist and classified as normal, atypical cells, HPV, mild, moderate and severe dysplasia, cancer *in situ* and invasive cancer. All abnormal smears as well as a 10% sample of normal smears were reviewed by two expert cytopathologists who re-classified the cytological diagnosis using the Bethesda system as normal, atypical squamous cells of undetermined significance, atypical glandular cells and LSIL or HSIL (Luff, 1992). The same pathologists read the biopsies and classified them as normal, cervicitis, cervical intraepithelial neoplasia (CIN) grade 1, 2, 3, carcinoma *in situ* or invasive cervical cancer.

### Statistical analysis

The outcomes of interest for this analysis were clearance, duration of incident type-specific HPV infections and incidence of CIN2/3 among women with normal cytology at enrolment. An incident type-specific HPV infection was defined as the first positive type-specific result after a negative result for the same HPV type. Correspondingly, type-specific HPV clearance was defined as the first negative PCR result after an incident infection. Duration of an HPV incident infection was defined as the elapsed time from the date of infection to the date of clearance, assuming both events occurred at the mid point between consecutive visits with different HPV status (i.e. negative to positive or positive to negative). Because women could have one or more HPV infections during follow-up, HPV type-specific infections, instead of individuals, were the unit of analysis. HPV infections were categorised using phylogenetic (de Villiers *et al*, 2004) and epidemiological classifications (Muñoz *et al*, 2003) in six groups as follows: HPV16, HPV18, *α*-9 HPV types other than HPV16 (i.e. HPV31, 33, 35, 52, 58, 67), *α*-7 HPV types other than HPV18 (i.e. HPV39, 45, 68, 70), other HPV-HR types and LR HPV types.

In the analysis of clearance and duration of infection, we censored women lost to follow-up after an incident infection and those with a gap longer than 36 months between consecutive visits because the most HPV infections clear within this time (Insinga, 2007). Also, women were censored if CIN2/3 was diagnosed.

Cox regression was used to evaluate factors potentially associated with clearance, including viral load, co-infection, age at infection, previous infection during follow-up, sexual behaviour, parity and tobacco use. Viral load was included in the analysis as the maximum viral load attained during an incident infection for each defined viral group. Because there is no consensus on reference values of ODs to classify viral load, they were categorised in quintiles. The Kaplan–Meier method was used to estimate the median duration of infection for most HPV types and for each of the previously defined viral groups. Infections were considered persistent if duration was above the median.

End points of interest for the CIN2/3 analysis included histology confirmed CIN2 (9), CIN3 or carcinoma *in situ* (14), invasive carcinoma (3) and cases of HSIL without histology confirmation (6). The latter group was included because of the high specificity of Pap smear and the low sensitivity of one colposcopically directed biopsy. A recent report indicates that one colposcopically directed biopsy misses at least one-third of CIN3+ (Gage *et al*, 2006).

To evaluate the association of our proposed definition of persistent infection with the risk of CIN2/3 development we carried out a Cox regression analysis adjusting for relevant cofactors of HPV infection (i.e. age, parity, OC use, smoking status and co-infection). Alternatively, we also used a longitudinal approach grouping all possible triplets of consecutive visits by individual to compare the results of the new approach with those obtained using the traditional definition of persistence (ie two consecutive positive visits) (Zeger and Liang, 1986). Specifically, HPV type-specific status was assessed at the first two visits of every triplet and classified as: (1) negative at both, (2) positive at either or (3) positive at both visits. Women should have normal cytology at the first visit of the triplet and lesion progression was assessed in the third. Here the time lag between visits was not restricted and logistic regression analysis was carried out using generalised estimating equations to account for the correlation between triplets of visits contributed by the same individual (Zeger and Liang, 1986). In both approaches, we considered incident and prevalent HPV infections and we adjusted for the same HPV cofactors.

## Results

Of the 2139 women who agreed to participate in the cohort study, 261 had only one visit during follow-up and 150 had abnormal cytology at baseline leaving 1728 (80%) women eligible for this analysis (154 included based on data from the second visit). This group represented 12 526 follow-up visits with cervical scrapes tested for HPV DNA.

The prevalence of HPV infection at baseline was 13.4% (224). During follow-up, 253 and 209 women HPV DNA negative at baseline developed single and multiple incident HPV type infections, respectively. In addition, 110 women who were HPV DNA positive at baseline developed incident infections (i.e. infections with a different HPV type) during follow-up. Thus, a total of 572 women developed 872 type-specific incident HPV infections during follow-up; 64 infections were excluded from the analysis because the interval between consecutive visits was longer than 36 months or got no further visits after the infection (Table 1). Median interval between visits was 6.6 months (interquartile range, IQR: 6.0–9.2), median duration of follow-up for clearance analysis was 6.4 years (IQR: 3.7–8.5 years) and median duration of total follow-up was 8.9 years (IQR: 6.3–9.4 years).

Maximum viral load attained during follow-up ranged from 0.18 to 4.07 OD. The IQR was similar among HR HPV types groups, with a median of 1.8–2.0 OD, whereas the median was lower in the LR HPV group (0.8 OD).

**Figure 1:**
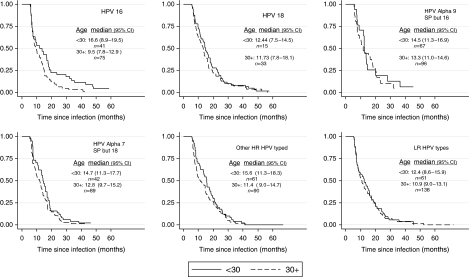
Cumulative probability of clearance of incident HPV infection, by viral group and age group (under 30 years and 30 years and over), in women with normal cytology at baseline. Number of infections (*n*) with point estimates and 95% CI of median duration of infection by age group are reported. HPV16 persisted significantly longer in women under 30 years of age than older women (*P*<0.01, log-rank test).

Cox regression analysis including all relevant cofactors for HPV infection shows that clearance of incident infections by HPV types is inversely associated with viral load (Table 1). The likelihood of clearance was significantly reduced for infections with higher viral load for all HPV groups (*P*-value for trend <0.001). With the exception of use of an intrauterine device that doubled the chance of clearance for HPV18 infections and age older than 30 years that increased 70% the probability of clearance of ‘other HR HPV’, none of the factors affected the likelihood of clearance. For all HPV groups, except HPV16, clearance was similar in women under 30 years of age and in women aged 30 years or older. Although no apparent differences in the probability of clearance between HPV groups were observed (Figure 1), difference of clearance with age in HPV16 infection (log-rank test *P*<0.01) was lost in the adjusted analysis (Table 1).

**Figure 2:**
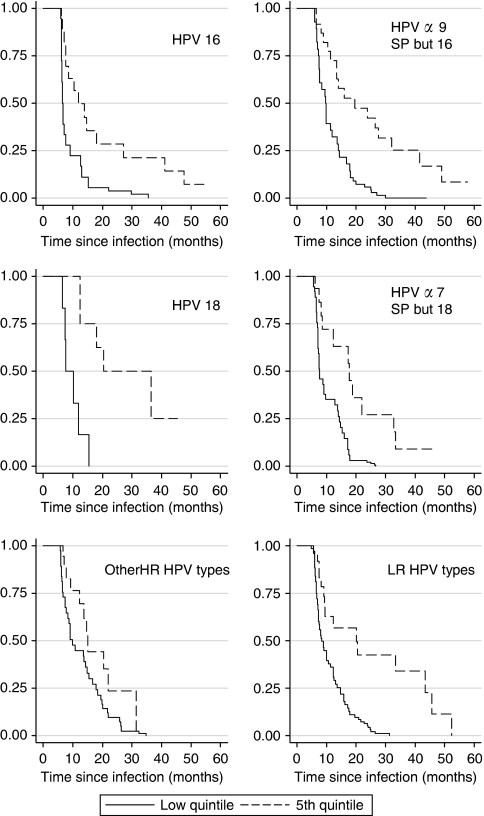
Cumulative probability of clearance of incident HPV infections by viral load (lower and fifth quintile) and viral group, in women with normal cytology at baseline. Viral load was estimated from the optical density and values categorised in quintiles.

Median duration of HPV incident infection seemed higher for HR HPV types than for LR types (Figure 1), but confidence intervals (CI) between types and between age groups had substantial overlap, except for HPV16 for which younger women had significantly longer persistence of infection. Although virtually all infections with lowest viral loads were cleared by the second year of follow-up, those with viral load in the highest quintile persisted longer. However, after 5 years of follow-up most of the infections have cleared (Figure 2).

Among women with normal cytology at baseline, 32 CIN2/3/HSIL incident cases were diagnosed during follow-up (0.23 per 100 person-years; 95% CI 0.16–0.33). Incident HPV infections occurring in women under age 30 carry a risk of CIN2/3 similar to that of incident infections occurring later (<30: 0.025 per 100 person-years, 95% CI 0.015–0.042; ⩾30: 0.023, 95% CI 0.014–0.037). Most cases (*n*=25) were associated with HPV16 and other types from the *α*-9 group. For HPV16, only those infections that lasted more than the median duration were associated with an increased risk of CIN2/3/HSIL, compared with no HPV16 infection, and this occurred in both age strata (<30 and ⩾30). The two cases of CIN2/3 that occurred after infection of short duration were diagnosed in the second visit.

For α-9 other than 16, infections lasting less than the median, as well as those lasting more, were associated with an increased risk of CIN2/3/HSIL (Table 2); this also applied in the analysis based on triplets. Risk of CIN2/3 was higher for HR HPV types at two consecutive visits than for infections positive at only one visit; no significant association was observed for LR types. For HPV16 the increased risk was particularly marked when women were positive in two consecutive visits and it was also increased, but not significantly, when women were positive in only one visit. For *α*-9 other than 16, an increased risk was observed for women positive at one of the two consecutive visits, as well as at the two consecutive visits. Median time between the first two visits was similar for all HPV group infections (7.5 months) (Table 3).

## Discussion

Understanding the natural history of HPV infection and the resulting cervical lesions requires long-term follow-up studies in which cervical cells and risk factor information are collected at frequent intervals to monitor HPV status and appearance of cervical lesions. Most studies (Ho *et al*, 1998; Moscicki *et al*, 2001; Woodman *et al*, 2001; Brown *et al*, 2005; Winer *et al*, 2005) include young women from populations at LR for cervical cancer and do not discriminate incident from prevalent infections in the analysis. Our study was conducted in a population at HR of cervical cancer, including young and older women, where incident HPV infections were considered separately. A high prevalence and incidence of cervical HPV infection has been reported in this cohort (Molano *et al*, 2002a; Muñoz *et al*, 2004).

Our results show that viral load is the main determinant of clearance and that clearance of incident infection occurs, in almost all cases, at 5 years showing no difference between viral types. This is remarkable considering possible misclassification in viral load measurements due to the semiquantitative method used in this cohort study. This method has shown moderate correlation with the real-time PCR assays that are considered more accurate (Hesselink *et al*, 2005). The importance of viral load as a determinant of persistence supports recent observations suggesting that GP5+/GP6+ viral load is a good parameter to distinguish those HR HPV infections that will progress to CIN3+ from those that will not (Snijders *et al*, 2006; Gravitt *et al*, 2007). Our results agree with other longitudinal studies using semiquantitative and quantitative PCR assays that show that high viral load is associated with an increased risk for HSIL or CIN2/3 (Ylitalo *et al*, 2000; van Duin *et al*, 2002; Schlecht *et al*, 2003; Dalstein *et al*, 2003; Monnier-Benoit *et al*, 2006). However, other studies using hybrid Capture 2 viral load measurement for a pool of 13 carcinogenic HPV types have yielded negative results (Lorincz *et al*, 2002; Castle *et al*, 2004). There are at least two possible explanations for the discordant results: first, comparison between studies is difficult because results are not given in absolute measurements; second, studies using hybrid Capture 2 viral load measurement provide an additive measurement of 13 types, whereas those giving positive results provide measurement for individual types and most have focused on HPV16. Although quantitative PCR techniques provide information on the amount of viral particles in each cell and correct for differences in amplification efficiency between samples, they cannot be used in large studies because they are laborious, expensive and have been developed only for a few HPV types (HPV16, 18, 31, 33, 45 and 58). In contrast, semiquantitative assays, though they analyse the number of viral copies by scrapes and not by cells and perform less efficiently in an intermediate viral load range, seem to perform adequately in high viral load ranges that are clinically relevant because they are associated with an increased risk of disease. However, one limitation of our findings is that we have used the maximum viral load attained during an incident infection and little is known on the dynamics of viral load during the course of an incident infection.

The lack of differences in persistence by viral type is at odds with the results obtained in a previous analysis of the same population where only prevalent HPV infections were considered (Molano *et al*, 2003). HPV16 had a significantly lower clearance than LR types and HR types other than *α*-9 species, and HPV types related to HPV16 had intermediate clearance rates. Such differences might be due to our restricting the analysis to incident infections in women with normal cytology at baseline and conducting a separate analysis by viral group instead of comparing between them. This approach gave us more observations to analyse and a straightforward interpretation of results as no viral type was considered as reference value.

An unexpected result was the lack of difference in the probability of clearance in women by age (under and over 30), contrary to a report from Guanacaste, Costa Rica that persistence increases with age (Castle *et al*, 2005). However, our results are in agreement with the lack of association between age and duration of infection recently reported from a cohort study similar to ours in Brazil (Trottier *et al*, 2008). Differences in study design (including the definition of persistence and inclusion of prevalent and incident HPV infections) and in statistical analysis may explain the discrepancies. In the Guanacaste study, persistence was defined as HPV DNA positivity at enrolment and at a single follow-up visit 5–7 years after enrolment, and survival analyses were not used.

We are proposing a new definition of persistence based on duration of incident infection, that is persistent infections are those lasting more than the median duration.

We believe that this definition is more informative than the traditional based on two consecutive HPV-positive tests that can be separated by irregular time intervals.

The risk pattern of CIN2/3/HSIL associated with the two definitions of persistence was remarkably similar. This is not surprising considering that our follow-up visits were separated by regular intervals of about 7 months. When persistence was defined as HPV DNA positivity at two consecutive visits, the risk was higher than when HPV DNA positivity was detected only at one of the two consecutive visits.

These results accord with those obtained in a recent meta-analysis in which persistence defined as HPV positivity at two or more time points was associated with an increased risk of CIN2/3 in most studies. However in this meta-analysis there was notable heterogeneity among the few studies that provided sufficient data to obtain HPV16 and 18 type-specific associations (Koshiol *et al*, 2008).

Our finding of an increased risk for CIN2/3 associated with HPV positivity at only one follow-up visit for *α*-9 types other than HPV16 indicates that HPV infections of short duration may also increase the risk of CIN2/3 lesions or that persistence is not essential for progression to high-grade lesions.

Our results accord with recent reports that HPV type and persistence are the main determinants of progression to CIN2/3. In particular, repeat detection of HPV DNA16 is associated with extremely high cumulative risk of subsequent CIN3+ diagnosis, exceeding 30% in some cohorts (Schiffman *et al*, 2007; Rodriguez *et al*, 2008).

Our study has several strengths, including the relatively large sample size, the broad age range covered, the low proportion of refusals, the long follow-up period, the short interval between follow-up visits (median 6.6 months), the comprehensive information collected at baseline and during follow-up on risk factors, and the use of sensitive and well-validated PCR assays for the detection of HPV DNA in a central laboratory. The main limitation of our study is the relatively small number of CIN2/3 lesions detected, which makes our risk estimates unstable.

In conclusion, in this cohort of Colombian women, viral load is the most important determinant of HPV persistence, and persistence of HPV16 infections carry a higher risk of CIN2/3. The occurrence of CIN2/3 after non-persistent *α*-9 infections other than HPV16 suggests that such lesions are early manifestations of HPV infection and are more likely to regress. These observations have important implications in forecasting the impact of prophylactic HPV vaccines and screening programs using HPV tests.

## Conflict of interest

Authors do not have a commercial or other association that may pose a conflict of interest.

## Figures and Tables

**Table 1:**
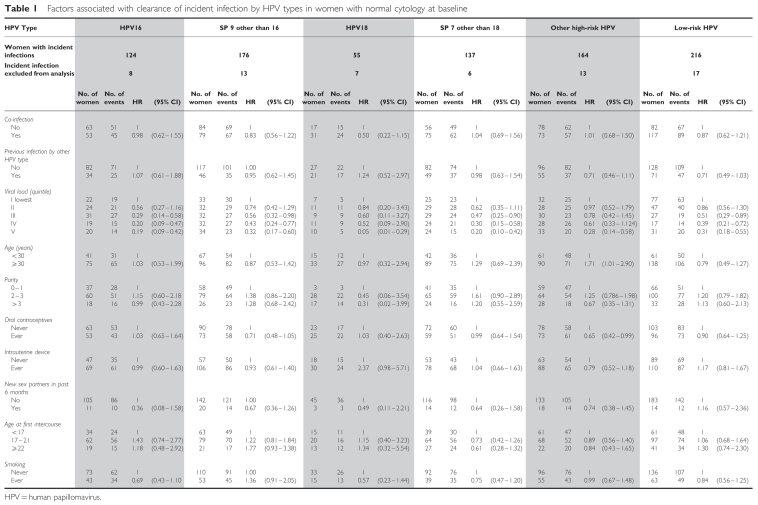
Factors associated with clearance of incident infection by HPV types in women with normal cytology at baseline

**Table 2:**
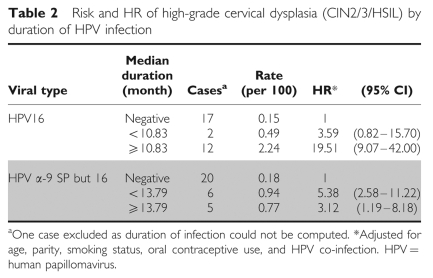
Risk and HR of high-grade cervical dysplasia (CIN2/3/HSIL) by duration of HPV infection

**Table 3:**
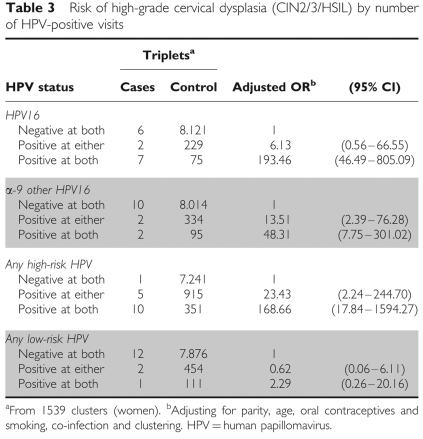
Risk of high-grade cervical dysplasia (CIN2/3/HSIL) by number of HPV-positive visits
